# A cross‐sectional natural history study of aspartylglucosaminuria

**DOI:** 10.1002/jmd2.12294

**Published:** 2022-07-14

**Authors:** Kimberly Goodspeed, Daniel Horton, Andrea Lowden, Peter V. Sguigna, Timothy Booth, Zhiyue J. Wang, Veronica Bordes Edgar

**Affiliations:** ^1^ Department of Pediatrics University of Texas Southwestern Medical Center Dallas Texas USA; ^2^ Children's Health Dallas Dallas Texas USA; ^3^ Department of Neurology University of Texas Southwestern Medical Center Dallas Texas USA; ^4^ Department of Psychiatry University of Texas Southwestern Medical Center Dallas Texas USA; ^5^ Department of Radiology University of Texas Southwestern Medical Center Dallas Texas USA

**Keywords:** AGA, aspartylglucosaminuria, electrophysiology, gene transfer therapy, lysosomal storage disorder, neuroimaging

## Abstract

Aspartylglucosaminuria (AGU) is a rare lysosomal storage disorder that causes stagnation of development in adolescence and neurodegeneration in early adulthood. Precision therapies, including gene transfer therapy, are in development with a goal of taking advantage of the slow clinical course. Understanding of disease natural history and identification of disease‐relevant biomarkers are important steps in clinical trial readiness. We describe the clinical features of a diverse population of patients with AGU, including potential imaging and electrophysiological biomarkers. This is a single‐center, cross‐sectional study of the clinical, neuropsychological, electrophysiological, and imaging characteristics of AGU. A comprehensive assessment of eight participants (5 Non‐Finnish) revealed a mean non‐verbal IQ (NVIQ) of 70.25 ± 10.33 which decreased with age (rs = −0.85, *p* = 0.008). All participants demonstrated deficits in communication and gross/fine motor dysfunction. Auditory and visual evoked potentials demonstrated abnormalities in one or both modalities in 7 of 8 subjects, suggesting sensory pathway dysfunction. Brain imaging demonstrated T2 FLAIR hypointensity in the pulvinar nuclei and cerebral atrophy, as previously shown in the Finnish AGU population. Magnetic resonance spectroscopy (MRS) showed a 5.1 ppm peak corresponding to the toxic substrate (GlcNAc‐Asn), which accumulates in AGU. Our results showed there was no significant difference between Finnish and Non‐Finnish patients, and performance on standardized cognitive and motor testing was similar to prior studies. Age‐related changes on functional assessments and disease‐relevant abnormalities on surrogate biomarkers, such as MRS, could be used as outcome measures in a clinical trial.

**Synopsis**

Aspartylglucosaminuria (AGU) is a slowly progressive lysosomal storage disorder with deficits in communication and motor dysfunction that can be quantified using standardized psychometric testing and clinical biomarkers such as brain imaging and magnetic resonance spectroscopy.

## 1 INTRODUCTION

Aspartylglucosaminuria (AGU) is an ultrarare, neurodegenerative lysosomal storage disorder (LSD), resulting in death by the sixth decade of life.^1^, ^2^ Due to a Finnish founder effect, there is a high carrier frequency of the Fin_Major_ (G482A and G488C) and Fin_Minor_ (two base pair deletion in exon 2) variants.^3^, ^4^, ^5^ Treatment for AGU is limited to supportive care, but the slow progression creates a large therapeutic window for disease‐modifying therapy.^6^ Early studies of bone marrow transplantation for AGU demonstrated slow biochemical rescue, however, long‐term follow‐up showed severe post‐transplant complications and worse developmental outcomes in comparison to untreated AGU patients, although transplantation during infancy shows more promise.^7^, ^8^, ^9^ Alternatively, a chaperone therapy to improve AGA enzymatic activity and gene transfer therapy remain promising options for future treatments.^10^, ^11^ To assess the efficacy of novel treatments, a deep understanding of the natural progression of AGU and the development of biomarkers are needed.

AGU is caused by biallelic *AGA* variants on chromosome 4q34.3. This results in deficiency of the enzyme aspartylglucosaminidase and toxic accumulation of *N*‐acetylglucosamines (GlcNAc‐Asn) within the lysosome.^12^ The detrimental effects are widespread, with profoundly negative effects on neuronal tissue.^13^, ^14^ Brain magnetic resonance imaging (MRIs) show nonspecific changes including poor gray–white matter differentiation, T2 hypointensities within the deep gray matter (e.g., caudate, putamen, thalamus), periventricular T2 hyperintensities, and T2 hypointensities within the corpus callosum.^15^, ^16^, ^17^, ^18^ A single case report of an adolescent with AGU revealed a smaller thalami and larger amygdala with progressive cerebral atrophy in comparison to a healthy twin.^18^ Thalamic T2 hypointensities are seen in other LSDs, but in AGU, it is most conspicuous within the pulvinar nucleus.^17^, ^18^, ^19^, ^20^, ^21^ Additional studies have identified corresponding signal hypointensity on susceptibility‐weighted imaging in the pulvinar nuclei, suggesting this may be due to iron deposition, as has been described in other LSDs.^22^, ^23^ This finding may correlate with the neuropsychological functioning of these individuals as the pulvinar nuclei play an important role in attention and executive functioning. Individuals with AGU are likely to have deficits in executive functioning and slowed processing speed.^24^, ^25^, ^26^, ^27^

Although the phenotype is well described in the Finnish population, clinical progression in non‐Finnish patients has not been adequately characterized and biomarkers of neurological functioning are limited. To address knowledge gaps of the effects of ethnic diversity on the natural clinical progression of AGU and to explore potential clinical outcome measures for future clinical trials, we conducted a cross‐sectional natural history study of a cohort of AGU patients of varied ethnic background and genotype.

## 2 PATIENTS AND METHODS

Participants were recruited to participate in a single‐center, cross‐sectional observation study of AGU. The study was posted on ClinicalTrials.gov (NCT03853876), and patients were referred by the patient advocacy groups (Rare Trait and Suomen AGU ry). The diagnosis of AGU was confirmed by review of a genetic testing report reflecting bi‐allelic pathogenic or likely pathogenic variants in the *AGA* gene. Participants completed a neurological history and physical, neuropsychological testing, motor assessments, electrophysiological measures, and brain and retinal imaging. Informed consent was obtained from the legal guardian of each participant and the study was approved by the institutional review board of the study site, the University of Texas Southwestern Medical Center.

### 2.1 Functional assessments

Participants completed the following neuropsychological measures in the following order based on the participant's age unless a deferment was specified: Leiter International Performance Scale, 3rd Edition (Leiter‐3; G. H. Roid, L. J. Miller, 2013), Receptive One‐Word Picture Vocabulary Test, 4th Edition (ROWPVT‐4; R. Brownell, 2010), Expressive One‐Word Picture Vocabulary Test, 4th Edition (EOWPVT‐4; R. Brownell, 2010), and the Vineland Adaptive Behavior Scales, 3rd Edition Comprehensive Interview (VABS‐3; S. S. Sparrow, D. V. Cicchetti, and C. A. Saulnier, 2016). Pediatric neuropsychologists administered the cognitive and behavioral testing (Veronica Bordes Edgar and Daniel Horton). Tests were selected to provide the broadest assessment of abilities across cultures. The assessment was limited to nonverbal tasks in non‐English‐speaking individuals except for the single‐word vocabulary tests that were administered with the assistance of certified medical interpreters. Participants were allowed to take breaks from testing when they showed signs of fatigue.

The participants completed the motor battery of the NIH Toolbox® for Assessment of Neurological and Behavioral Function—Motor Battery (NIH Toolbox) as well as the 6‐min walk test (6MWT). NIH Toolbox assessments include the 9‐hole Pegboard Dexterity and Standing Balance tests. The 6MWT was administered by an experienced physical therapist, and the NIH Toolbox Motor Battery and neuropsychological assessments were administered by pediatric neuropsychologists (Veronica Bordes Edgar and Daniel Horton).

### 2.2 Electrophysiological assessments

We utilized pattern‐reversal and flash visual evoked potential (VEP). Flash VEP was used in participants who were unable to fixate and was performed using flash goggles with recording parameters of a timebase of 250 ms, filter settings of 1–100 Hz, and repetition rate of 2.11/s. Pattern‐reversal VEP was performed using a black and white checkerboard pattern‐reversal monitor presented monocularly at a distance of 70 cm from the participant with recording parameters of a timebase of 200 ms, filter settings of 1–100 Hz, and repetition rate of 1.05/s. The latency and amplitude of the first positive peak (p100) was measured in response to the visual stimulus for participants able to complete the pattern‐reversal VEP. A qualitative report of absence or presence of the p100 is available for participants who completed the flash VEP. The VEP was performed by a trained neurophysiology technician and interpreted by a single reader trained in clinical pediatric neurophysiology (Andrea Lowden).

Brainstem auditory evoked response (BAER) was performed using rarefaction click stimuli at 70 dB with recording parameters including a timebase of 15 ms, filter settings of 30–3000 Hz, and repetition rate of 11.1/s. Three hundred sweeps were performed for each ear with the contralateral ear masked at 45 dB. The BAER was performed by a trained neurophysiology technician and interpreted by a single reader trained in clinical pediatric neurophysiology (Andrea Lowden).

### 2.3 Imaging

Optical coherence tomography (OCT) and blue light autofluorescence imaging were performed using the Spectralis OCT, as previously described.^21^, ^28^ All scans were obtained by a single operator under low‐light conditions without mydriasis. Peripapillary scans and macular scans of a 3 × 3 mm area by high‐speed protocol were acquired as tolerated. Both peripapillary and macular scans underwent semi‐automated segmentation of the retinal nerve fiber layer (RNFL) and macular volume (MV). Manual correction of the segmentation was performed as required following review of acquisition. MV volume was taken as the measurement across a standard 6 mm diameter Early Treatment in Diabetic Retinopathy Study (ETDRS) grid. Interpretation was performed by a single reader (PS). Quality standards were defined as per the OSCAR‐IB criteria.^29^ Data were reported in accordance with APOSTEL recommendations.^30^

The MRI/magnetic resonance spectroscopy (MRS) was performed on a 3T clinical MRI scanner (Skyra, Siemens Healthliners) at Children's Medical Center Dallas. In the same MRI/MRS session, T1‐weighted 3D MPRAGE, T2 weighted 3D FLAIR and diffusion tensor imaging was acquired. MRIs were interpreted by radiologists with specialty training in neuroradiology. For MRS, a single voxel spin‐echo sequence was used (TR/TE = 2000/135 ms, voxel 2 × 2 × 2 cm^3^, number of signal average = 128, water suppression bandwidth = 20 Hz). The region of interest (ROI) was placed on the left thalamus (Zhiyue J. Wang). Pulse sequence was tested using a solution phantom containing *N*‐acetyl‐d‐glucosamine (GlcNAc)^31^ because GlcNAc‐Asn was unavailable. In the α‐GlcNAc there is a peak at 5.2 ppm which is 0.4 ppm away from the water peak at 4.8 ppm at room temperature and 4.7 ppm at body temperature, therefore, the phantom reasonably mimics the in vivo situation. We reduced the bandwidth of the water suppression pulse from 50 to 20 Hz to avoid excessive saturation of the 5.1 ppm signal.

### 2.4 Statistical analyses

Because this was an exploratory study with a small study population in a rare disorder, we used descriptive statistics and nonparametric tests for comparisons and correlations except where assumptions of normality were met. The Spearman correlation coefficient was used to evaluate the relationship between age and nonverbal IQ (NVIQ), expressive vocabulary skills (EOWPVT‐4 standard scores), receptive vocabulary skills (ROWPVT‐4 standard scores), and adaptive functioning (VABS‐3). The point biserial correlation was used to compare the results of the BAER to ROWPVT‐4 and VEP to Beery VMI. A one‐sample *t*‐test was used to compare the nonverbal IQ scores to a standard population mean of 100. A paired *t*‐test was used to compare the expected versus the actual distance traveled by participants on the 6MWT. The data analysis was generated using SAS software, Version 9.4 of the SAS System for the University of Texas Southwestern.

## 3 RESULTS

### 3.1 Demographics

Eight participants were enrolled in the study (Table 1). The mean age of the participants at the time of the study visit was 14.48 years (SD 5.37). There were 4 males (2 sibling pairs) and 4 females. Finnish patients had both Fin_Minor_ and Fin_Major_ variants, while non‐Finnish patients had microdeletions, missense, and nonsense variants. Non‐Finnish subjects were of Asian, African, and European decent.

**TABLE 1:** Demographics**—**participant's gender, age at the time of evaluation, country of origin, and genotype are detailed

Subject ID	Gender	Age at evaluation (year)	Allele 1	Allele 2	Race ethnicity
Non‐Finnish					
1	M	24.42	Deletion (34 kb)	Thr122Lys	Not‐Hispanic White
2	M	19.25	Deletion (34 kb)	Thr122Lys	Not‐Hispanic White
6	F	11.58	Arg107^a^	Glu340^a^	Not‐Hispanic Black
7	F	10.58	c.128‐2A>G (Intronic splice site alteration)	c.128‐2A>G (Intronic splice site alteration)	Not‐Hispanic Asian
8	F	16.83	p.S72P	p.W168X	Not‐Hispanic White
Finnish					
3	F	14.25	Glu67fsX3^b^	Glu67fsX3^b^	Not‐Hispanic White
4	M	10.08	Cys163Ser^c^	Cys163Ser^c^	Not‐Hispanic White
5	M	8.83	Cys163Ser^c^	Cys163Ser^c^	Not‐Hispanic White

*Notes*: The Fin_Minor_ Variant (G482A and G488C) is represented as Cys163Ser as this is believed to cause enzymatic deficiency. Participants 1, 2, 7, and 8 have been included in prior publications.^10^, ^21^, ^32^, ^33^

^a^ Non‐sense variant.

^b^ Fin_Minor_ variant.

^c^ Fin_Major_ variant.

### 3.2 Functional assessments

The nonverbal IQ (NVIQ) for the group ranged from 56 to 83 (mean 70.25, ±10.33), falling in the low average to exceptionally low range (Table 2).^34^ The VABS‐3 revealed scores well below the standard mean of 100 on all composite and subdomains, and all participants showed deficits in communication abilities with scores in the low to exceptionally low range of <55–79 on the EOWPVT‐4 and < 55–117 on the ROWPVT‐4. The mean age‐equivalent on the EOWPVT‐4 was 6.4‐years (±1.7‐years) and 8.1‐years (±4.4‐years) on the ROWPVT‐4. There was a strong negative correlation between age and NVIQ as well as adaptive composite standard score (age*NVIQ rs = −0.85, *p* = 0.008; age*adaptive composite standard score rs = −0.85, *p* = 0.007). There was not a significant correlation between age and the sum of the raw scores on the sub‐domains of the VABS‐3 or expressive/receptive language skills. There was no significant difference in the mean NVIQ or VABS‐3 adaptive composite scores by gender or Finnish versus Non‐Finnish. When accounting for age, height, and weight, the mean difference between expected and actual distance traveled was significantly reduced (−186.2 m ± 71.1, *p* = 0.0001).^35^, ^36^ None of our participants had significant cardiac or respiratory disease. Participants 2 and 3 have scoliosis. Participants scored ≤5th percentile on total time to completion of the peg‐board task with the dominant hand, and ≤ 6th percentile with the non‐dominant hand. On the balance assessment, the three youngest participants scored in the 38–80th percentile (less sway) while the remainder of the participants scored ≤6th percentile (more sway).

**TABLE 2:** Functional assessments**—**The non‐verbal IQ was calculated from the Leiter‐3

Subject ID	NVIQ	Expressive EOWPVT‐4	Receptive ROWPVT‐4	Adaptive composite	6MWT Distance (%E) Ht (cm), Wt (kg)	Balance score (%ile)	Peg Board Time to complete seconds (%ile)
Non‐Finnish							
1	57	58	76	25	577 (79.5) 185 cm, 73 kg	−0.427 (3)	85.25 (1)
2	56	95	97	45	510 (70.3) 178 cm, 64 kg	−0.345 (3)	50.42 (1)
6	83	84	89	71	505 (76.3) 149 cm, 44 kg	−0.963 (6)	39.99 (<1)
7	82	60	68	73	400 (60.4) 144 cm, 58 kg	−0.36 (38)	37.95 (1)
8	64	80	77	50	422 (63.5) 150 cm, 53 kg	−1.302 (1)	33.13 (1)
Finnish							
3	73	78	117	59	473 (71.3) 150 cm, 66 kg	−1.126 (1)	34.17 (1)
4	74	64	66	50	440 (65.4) 138 cm, 33 kg	−0.345 (38)	36.19 (2)
5	73	79	77	64	537 (92.9) 139 cm, 33 kg	0.154 (80)	32.72 (4)

*Notes*: Standard scores on the EOWPVT‐4, the ROWPVT‐4, and VABS‐3 adaptive composite are presented. Across all tests, scores fell approximately 1 to 3 SD below the standard mean, with a relative strength in receptive language abilities. Gross motor (6MWT), balance, and fine motor (peg board) are presented and demonstrated deficits in all areas. 6MWT is reported as the total distance traveled and percent expected for age, weight, and height (%E).

Abbreviations: EOWPVT‐4, Expressive One‐Word Picture Vocabulary Test, 4th Edition; NVIQ, non‐verbal IQ; ROWPVT‐4, Receptive One‐Word Picture Vocabulary Test, 4th Edition; VABS‐3, Vineland Adaptive Behavior Scales, 3rd Edition Comprehensive Interview; 6MWT, 6‐min walk test.

### 3.3 Clinical biomarkers

#### 3.3.1 Brain imaging

**FIGURE 1:**
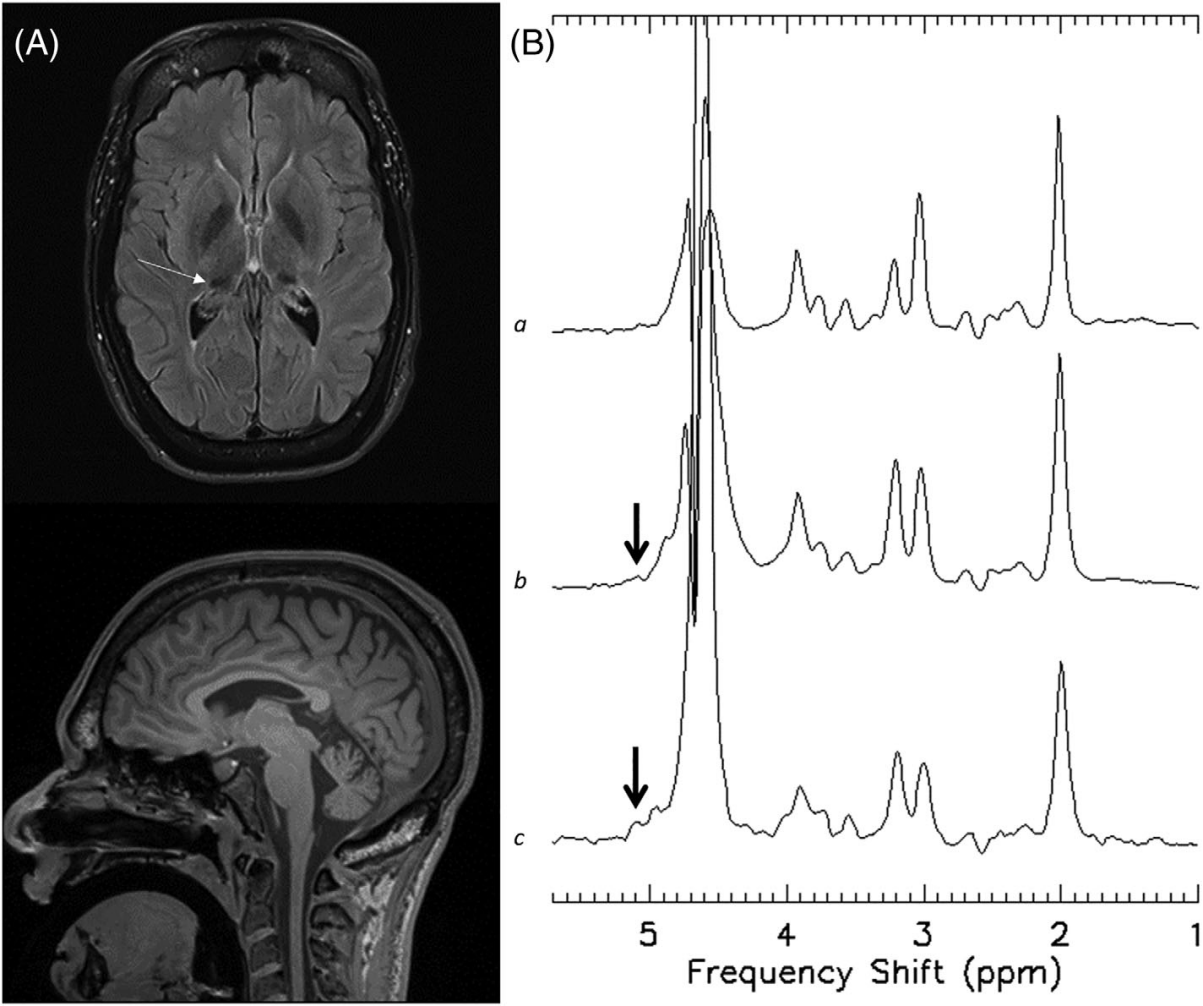
Brain magnetic resonance imaging (MRI) and magnetic resonance spectroscopy (MRS). (A) MRI Brain of subject 1 demonstrating T2 FLAIR hypointensity of the pulvinar nuclei (arrow) and cerebral/cerebellar atrophy. (B) MRS demonstrating comparison of a single healthy control (*a* top), average of spectral peaks of all subjects (*n* = 8) (*b* middle), and a single patient with aspartylglucosaminuria (AGU) (*c* bottom) showing the presence of a spectral peak at 5.1 ppm

Brain MRIs were abnormal in all participants and demonstrated T2 hypointensity within the pulvinar nuclei as previously reported, in addition to non‐specific T2 hyperintensities within the supratentorial white‐matter (7/8), loss of gray‐white matter differentiation (5/8), and cerebral atrophy (4/8) (Table 3, Figure 1).^20^ Cerebellar atrophy did not correlate with age. Single voxel MRS with the ROI over the thalamus achieved satisfactory B_0_ shim and water suppression in all participants and no baseline correction was performed. The peak of interest at 5.1 ppm was identified in 3/8 participants. Due to variability in peak height between participants, the average of the spectra from all participants demonstrated a smaller, but still discernible, peak at 5.1 ppm that potentially corresponds to GlcNAc‐Asn. MRS from a single control subject (ROI in the occipital lobe) did not demonstrate the peak at 5.1 ppm (Figure 1).

**TABLE 3:** Biomarker Assessments—Brain MRIs demonstrated abnormalities similar to previously reported studies in Finnish AGU patients

Subject ID	MRI brain	BAER	VEP		OCT	
			Right p100 (seconds)	Left p100 (seconds)	Right RNFL (μm)	Left RNFL (μm)
Non‐Finnish						
1	WM T2 hyperintensities, cerebellar and cerebral atrophy, T2 hypointensity in pulvinar nucelus	Prolonged latency R I‐V IPL	Absent	Absent	Incomplete	117
2	Periventricular T2 hyperintensity, cerebellar atrophy, ventriculomegaly, thickened calvarium, T2 hypointensity in pulvinar nucelus	Prolonged latency L I‐III IPL	90.3	83.7	113	111
6	Decreased GM‐WM differentiation, T2 hypointensity in pulvinar nucelus	Normal	95.3	96.4	106	92
7	WM T2 hyperintensities, decreased GM‐WM differentiation, periventricular T2 hyperintensity, thalamic atrophy with T2 hypointensity in pulvinar nucelus	Normal	113.6	109.3	99	93
8	WM pallor, periventricular T2 hyperintensity, T2 hypointensity in pulvinar nucelus of thalami, GP, red nucleus, SNg	Prolonged Latency: R Peak III	86.7	88.6	115	111
Finnish						
3	Periventricular T2 hyperintensity, T2 hypointensity in pulvinar nucleus of thalamus, decreased GM‐WM differentiation, cerebellar atrophy	Latency Asymmetry: R Peak V, R I‐V IPL, R III‐V IPL; Amplitude Asymmetry: R	Absent	Absent	107	102
**4**	Decreased GM‐WM differentiation, periventricular T2 hyperintensity, T2 hypointensity within pulvinar nucleus of thalamus	Decreased IV‐V/I amplitude bilaterally	Absent	Absent	Incomplete	Incomplete
**5**	Ventriculomegaly, Cerebellar atrophy, Decreased GM‐WM differentiation, T2 hypointensity in pulvinar nucelus of thalami, periventricular T2 hyperintensity	Prolonged latency: L Peak III, L I‐III IPL; Latency Asymmetry: L I‐III IPL	Absent	Absent	100	99

*Note*: To assess the integrity of the visual and auditory pathways, brainstem auditory evoked responses and visual evoked potentials are presented. All but one participant had abnormalities of one or both of these studies, suggesting dysfunction of these sensory pathways.

Abbreviations: BAER, brainstem auditory evoked response; GM, gray matter; GP, globus pallidus; IPL, inter peak latency; L, left; R, right; MRI, magnetic resonance imaging; OCT, optical coherence tomography; RNFL, retinal nerve fiber layer; WM, white matter; SNg, substantia nigra; VEP, visual evoked potential.

#### 3.3.2 Electrophysiological assessments

The BAER revealed abnormal results in six of eight participants including prolonged latencies, latency asymmetry, and decreased or asymmetric amplitudes. Prolonged latencies were both prolonged interpeak or absolute latencies and were identified over the right or left side. The VEP revealed abnormal results in five of eight participants. Of those with abnormal results, the p100 peak was not visualized in four of five. Flash VEP was used in these four due to inability to fixate on the stimulus. One participant had a delayed p100 peak at 113.6 s on the right and at the upper limit of normal at 109.3 s on the left using pattern reversal VEP. There were no technical or patient factors identified to account for the prolonged latency. All three Finnish participants revealed abnormal findings on both the BAER and flash VEP. There was no statistically significant relationship between abnormalities on either BAER or VEP with language abilities (ROWPVT‐4) or visual‐motor function (Beery VMI), respectively.

#### 3.3.3 Optical coherence tomography

OCT fulfilled at least five of seven OSCAR‐IB criteria for five of eight participants; partial studies were completed in three subjects due to technical difficulties. There was significant RNFL asymmetry in two participants, which was not congruous with MV asymmetry. Auto‐fluorescent inclusion bodies were observed in two participants (one previously reported and finding was stable).^21^ The second patient identified with auto‐fluorescent inclusion bodies was the oldest Finnish patient. The p100 was absent on flash VEP for both participants. Five of the six without auto‐fluorescent inclusion bodies displayed interocular RNFL asymmetry concerning for structural disease of the anterior visual system.

## 4 DISCUSSION

This comprehensive, cross‐sectional study of a genotypically and ethnically diverse cohort of participants with AGU expands upon the characterization of the Finnish AGU population and explores novel biomarkers of AGU (i.e., MRS, electrophysiological testing, and retinal imaging). Although this study is limited by a small sample size, it is the most diverse cohort of AGU patients reported to date. Because most studies of AGU are on the genotypically homogenous Finnish population, the generalizability was unclear. We have representation of both the Finn_Major_ and Finn_Minor_ variants as well as eight additional variants and three additional ethnicities (i.e., European‐Non‐Finnish, African, Asian). Because this was a diverse population, another potential limitation were language barriers and travel requirements, but study procedures were optimized for these anticipated challenges in the design phase.

We demonstrated cognitive deficits and brain imaging findings (i.e., thalamic T2 hypointensity, cerebral atrophy) similar to those previously published in the Finnish AGU population, suggesting loss of the AGA enzyme activity, regardless of genotype, leads to similar patterns of neurodegeneration. Although the sample size was not adequately powered to measure the impact of ethnicity or genotype on cognitive abilities, there was no difference in NVIQ between Finnish and non‐Finnish subjects. To account for language and cultural differences, we used the Leiter International Performance Scale, 3rd Edition to assess global cognitive functioning, which does not generate a full‐scale IQ (FSIQ).^37^ Although our NVIQ scores were higher than full‐scale IQ (FSIQ) scores in prior Finnish studies, our NVIQ scores were consistent with the non‐verbal subtests among the Finnish AGU studies.^38^ This suggests that the FSIQ may be negatively impacted by the verbal IQ scores. This assertion is supported by the low age‐equivalent scores on the EOWPVT‐4 and the ROWPVT‐4, suggesting verbal skills, especially expressive communication abilities, are weak in AGU. The VABS‐3 Adaptive Composite score, a caregiver‐report measure that correlates to cognitive functioning, was one standard deviation lower than the NVIQ scores. This discrepancy typically indicates that other factors beyond low intellectual ability are impacting a patient's ability to meet the demands of daily life, such as deficits in higher‐order processes like attention and executive functioning. It may also reflect reduced parental expectations regarding self‐care and independent living skills, which may be impacted by cultural or genotypic differences. Finally, there was a decline in NVIQ with age, although there was only a small decrease in raw scores on the sub‐domains. The relative stability of the raw scores suggests there is a stagnation in the acquisition of new skills, but an increasing gap between expected age‐appropriate skills and chronological age.

Progressive motor impairments and fatigue were also described in the Finnish AGU population.^2^ Similarly, our cohort walked shorter distances on the 6MWT, demonstrated an age‐related impairment in balance, and had impaired fine motor skills. Deficits in motor function can be powerful outcome measures to include in a clinical trial because they can be repeated multiple times within the boundaries of clinical trial with less concern for improvement solely as a practice phenomenon and can translate to meaningful improvement in a patient's quality of life. We demonstrated that a diverse group of patients with AGU could perform the motor measurements included in this study, and that there is room for demonstrable improvement across all measures. Thus, these could be meaningful outcome measures to include in a clinical trial.

Because the rate of change on functional assessments can be slow, clinical biomarkers that detect a biochemical change on a shorter time scale would be valuable. We explored potential biomarkers including retinal imaging, electrophysiological testing, and brain MRS for surrogate measurement of substrate accumulation. Although most OCT, VEP, and BAER studies demonstrated abnormalities, the findings were heterogenous and suggest multifocal disease of the visual and auditory pathways. Results suggest that while there are quantifiable structural and functional abnormalities of these pathways, our findings (1) are not symmetric within individual patients, (2) are not uniform or specific to this disorder, and (3) did not always correlate with the age of the patient. This degree of heterogeneity limits the ability to perform group‐level analyses and would not likely be robust biomarkers in a clinical trial. In vivo measurement of GlcNAc‐Asn on MRS, however, would be specific to AGU and has the potential to demonstrate target engagement within the brain. We showed that in vivo detection of a 5.1 ppm peak by MRS was feasible in individuals with AGU. This peak could represent the toxic substrate of AGU and warrants further exploration in a larger cohort of AGU patients and healthy controls are needed. In addition, concurrent quantification of GlcNAc‐Asn by high‐field, high‐resolution NMR spectra in blood and urine of AGU patients would strengthen the interpretation of this disease‐specific biomarker. Establishing reliable quantification of GlcNAc‐Asn in blood, urine, and CNS will be powerful surrogate biomarkers in future clinical trials of precision therapies in AGU. For example, in the pre‐clinical studies of gene replacement therapy for AGU, enzyme levels were restored and substrate levels were reduced within 4 weeks of treatment, preceding the behavioral rescue.^11^ Thus, elimination of the GlcNac‐Asn peak at 5.1 ppm by MRS could be a meaningful early marker of efficacy in a clinical trial and may precede rescue of a functional outcome measures, as was demonstrated in the preclinical studies.

## 5 CONCLUSION

AGU is an ultrarare disease with new therapies under development. Although the NVIQ in our cohort was higher than previously published FSIQ, our data support the natural progression described in the Finnish population. Furthermore, analysis of the raw scores on cognitive testing demonstrates a plateau in abilities and could support the hypothesis that there is a large therapeutic window in AGU. We also demonstrated feasibility of functional motor assessments in a diverse cohort of AGU patients, which could be incorporated into a future clinical trial. Finally, while the findings on the clinical biomarker assessments demonstrate the feasibility to noninvasively detect deficits in the auditory and visual pathways, these findings were heterogenous and do not appear to have an AGU‐specific pattern. Conversely, biochemical measurement of the toxic substrate in AGU by MRS is promising and would be valuable in a clinical trial as it provides a way to noninvasively assess early target engagement in the brain. The combination of the functional assessments and clinical biomarkers explored in this study have the capability of demonstrating biochemical and functional rescue if properly incorporated into a future clinical trial of a precision therapy for AGU.
